# A data-driven estimation of the ribosome drop-off rate in *S. cerevisiae* reveals a correlation with the genes length

**DOI:** 10.1093/nargab/lqae036

**Published:** 2024-04-18

**Authors:** Sherine Awad, Angelo Valleriani, Davide Chiarugi

**Affiliations:** Genomics and Bioinformatics Core Facility, Institute of Metabolic Science, University of Cambridge, Cambridge, CB2 0QQ, UK; Max Planck Institute of Colloids and Interfaces, Science Park Golm, 14424 Potsdam, Germany; Max Planck Institute for Human Cognitive and Brain Sciences, Stephanstraße 1a, 04103 Leipzig - Germany

## Abstract

Ribosomes are the molecular machinery that catalyse all the fundamental steps involved in the translation of mRNAs into proteins. Given the complexity of this process, the efficiency of protein synthesis depends on a large number of factors among which ribosome drop-off (i.e. the premature detachment of the ribosome from the mRNA template) plays an important role. However, an *in vitro* quantification of the extent to which ribosome drop-off occurs is not trivial due to difficulties in obtaining the needed experimental evidence. In this work we focus on the study of ribosome drop-off in *Saccharomyces cerevisiae* by using ‘Ribofilio‘, a novel software tool that relies on a high sensitive strategy to estimate the ribosome drop-off rate from ribosome profiling data. Our results show that ribosome drop-off events occur at a significant rate also when *S. cerevisiae* is cultured in standard conditions. In this context, we also identified a correlation between the ribosome drop-off rate and the genes length: the longer the gene, the lower the drop-off rate.

## Introduction

Protein synthesis is one of the fundamental biochemical processes that characterise living organisms. A crucial step of this complex process consists of the translation of the messenger RNA (mRNA), where the information specified by the nucleotide sequence composing the mRNA template is decoded into the corresponding amino acids chain. Ribosomes are the molecular machinery that operates mRNA translation and allows it to proceed accurately and efficiently (1,2). Despite the activity of the ribosomes, protein synthesis can be subject to different kinds of errors that might lead either to amino acid misincorporations (3) or to the premature termination of the translation process (4,5). In some cases, translation abortion is a regulated mechanism that intervene to resolve stalled ribosomes (6), an event that occurs mainly when the cell faces stressing conditions that hinder mRNA translation such as, for example, amino acid starvation (7) or the local depletion of ternary complexes (8). In bacteria, the tmRNA-SmpB complex (9,10), RF3 (11) , ArfA (12) and ArfB (13) are main abortion-mediating factors that rescue stalling ribosomes and eventually lead to the premature termination of the protein synthesis. A proofreading mechanism is also known to interrupt the synthesis of miscoded polypeptides (14). In other cases, are the features of the mRNA template that trigger the translation abandonment as it happens when the nucleotide sequence includes ‘false’ stop codons (15,16).

Premature translation abandonment could also be the consequence of unspecific events, generally referred to as *processivity errors* (17,18). These are errors that occur during the translation process that are not related to the specific sequence that is currently translated and not dependent on the sequence of the protein being synthesized. Since the ribosome, as a molecular machine, undergoes a series of complex conformational changes during the elongation process, any such change can turn into an irreversible mistake. These mistakes taken together are called ‘processivity errors’ when they lead to a stop of translation and a drop-off of the ribosome from the mRNA template. The drop-off rate represents an unspecific processivity error, dependent on the inner machinery of the ribosomes more than on the specific sequence that the ribosome is translating. There are known sequences that induce a drop-off of the ribosomes, which are some kind of premature termination to allow different transcripts using the same mRNA sequence (short or long). These mechanisms are very rare though and they are unlikely to have an influence at the translatome level.

All the processes described so far lead eventually to the detachment of the ribosome from the mRNA template before it reaches the stop codon. Following (5), we will define ‘*ribosome drop-off*’ all these events, independently on the underlying mechanism.

Ribosome drop-off is not expected to occur only when the cell faces stressing conditions. Rather, it is reasonable to assume that a ‘basal’ drop-off rate can be associated with a non stressing environment.

Interestingly, following the seminal works of Kurland *et al.* (reviewed in (19)), some studies reported on ribosome drop-off and investigated its magnitude and dynamics. In (18,20,21), ribosome drop-off was clearly detected and estimated for the β-galactosidase gene through different *in vitro* approaches. In (22), an *in vivo* experiment estimated the drop-off rate for *E. coli* to be 4 × 10^−4^ events per codon. Noticeably, in (23), theoretical arguments demonstrate that the presence of a basal drop-off rate leads *necessarily* to an exponential distribution of the ribosomes density along the Coding Sequence (CDS) of the translated mRNA when the initiation rate of translation is the limiting rate. Interestingly, theoretical arguments have also shown that when the initiation rate is larger than the termination rate, the distribution of ribosomes along the mRNA in the presence of drop-off can become very different from a negative exponential (24).

In (5), the authors provided a quantitative method to estimate the ribosome drop-off rate through the analysis of Ribosome-profiling (Ribo-seq) data and used it to characterise protein synthesis abortion in *Escherichia coli*. Surprisingly, the measurements obtained in that work turned out to be in the range of 10^−4^ events per codon, thus reflecting the results previously obtained *in vivo* (22).

As discussed in (25), Ribosome drop-off has a clear impact on the dynamics of protein synthesis and an accurate quantitative estimate of the drop-off rate should be carefully considered when modelling ribosomes traffic and protein synthesis (26,27). Noteworthy, a better understanding of the quantitative aspects of premature mRNA translation termination could shed light on the mechanisms that regulate the efficiency of protein synthesis (28) with straightforward follow-ups in the biomolecular engineering domain such as, for example, the optimisation of the design of protein expression vectors for gene therapy or, more in general, for the production of recombinant proteins. The occurrence of ribosome drop-off could also be related to some interesting experimental observations about the mRNA translation process such as the relationships between the length of the Coding Sequences (CDS) and the corresponding protein abundance (29,30) or the relationship between CDS length and ribosomes density (26,31,32).

These arguments, together with the results obtained for *E. coli* in (5) motivated us to further investigate ribosome drop-off from a quantitative perspective in a more complex organism. In this work we study the features of ribosome drop-off in *Saccharomyces cerevisiae* through a data-driven computational approach. To this aim we developed Ribofilio, a software tool that allows effective quantification of the ribosome drop-off rate from the analysis of Ribo-seq data.

We show that while the ribosome drop-off rate is, overall, still in the range of 4 × 10^−4^ events per codon, the dynamics of ribosome drop-off in *S. cerevisiae* is significantly different from *E. coli* (5), reflecting the higher complexity of both the eukaryotes genome and its expression. Relying on Ribofilio, we characterise the possible drivers of this difference and we identify an interesting correlation between the genes length and the ribosome drop-off rate .

## Materials and methods

### The analysed datasets

In (32) Ingolia and the coauthors describe one of the most used experimental protocols for Ribo-seq and use it for the profiling of *S. cerevisiae* in log-phase growth on a rich medium. Interestingly, the study involved also cells cultured in conditions of acute amino acids starvation to understand how the ribosome profiles reflected the dynamics of the translation process in nutrients restricted media. The data produced in (32) offered us the chance to estimate the ribosome in drop-off rate in *S. cerevisiae* growing in a standard environment and to compare it with the corresponding rates determined when the cells are grown in a stressing environment. Indeed, we used the Ribofilio pipeline to analyse the datasets referred to as *D*5, *D*6, *D*7, and *D*8 in Table 1, obtaining the results reported in Table 2.

**Table 1.** Essential features and references of the analysed datasets

Dataset ID	GEO series	GEO sample ID: Ribo-seq	GEO sample ID: RNA-seq	Culture medium
**D1**	GSE91068	GSM2420488	GSM2420486	Synthetic Defined (SD)
**D2**	GSE134152	GSM3938059	GSM3938057	Synthetic Defined (SD)
D3	GSE91068	GSM2420489	GSM2420487	Methionine Restricted
D4	GSE134152	GSM3938060	GSM3938058	Glucose Restricted
**D5**	GSE13750	GSM346111	GSM346117	Rich (YEPD) (1)
**D6**	GSE13750	GSM346114	GSM346118	Rich (YEPD) (2)
D7	GSE13750	GSM346115	GSM346120	Amino Acid Starvation (1)
D8	GSE13750	GSM346116	GSM346122	Amino Acid Starvation (2)

Column 1: dataset’s reference ID, used in this paper; the IDs of the datasets used as reference in the comparative analysis (*S. cerevisiae* cultured in standard conditions) are highlighted in bold. Column 2: GSE series number. Column 3: GSM ID of the Ribo-seq data. Column 4 is the GSM ID of the corresponding mRNA data. Column 5: culture medium.

**Table 2.** Ribosome drop-off rates estimated by Ribofilio (bin-size = 50)

Dataset	Drop-off rate per bin (*r*_*b*_)	Drop-off rate per codon (*r*_*c*_)	RMSE	$\boldsymbol{R^2}$	SE	CI	p-value
**D1**	0.0051	0.0003	0.0143	0.4907	0.0006	*r*_*b*_ ± 0.0011	<0.00001
**D2**	0.0102	0.0006	0.025	0.6887	0.0007	*r*_*b*_ ± 0.0013	<0.00001
D3	0.0100	0.0006	0.0762	0.4104	0.0011	*r*_*b*_ ± 0.0021	<0.00001
D4	0.0073	0.0004	0.0282	0.4993	0.0003	*r*_*b*_ ± 0.0006	<0.00001
**D5**	0.0057	0.0003	0.4843	0.0341	0.0021	*r*_*b*_ ± 0.0041	0.0038
**D6**	0.0053	0.0003	0.3147	0.0462	0.0019	*r*_*b*_ ± 0.0038	0.0029
D7	0.0079	0.0005	0.5336	0.0594	0.0027	*r*_*b*_ ± 0.0053	0.0016
D8	0.0077	0.0005	0.4188	0.0709	0.0027	*r*_*b*_ ± 0.0052	0.0020

Column 1: Dataset ID (Table 1); the datasets used as reference in the comparative analysis (*S. cerevisiae* cultured in standard conditions) are highlighted in bold. Column 2: Drop-off rate per bin (rb). Column 3: Drop-off rate per codon (rc). Column 4: RMSE. Column 5: coefficient of determination (R2). Column 6: Standard Error Estimate (SE).Column 7: Confidence Interval 95%. Column 8: *P*-value resulting from the *t*-test (null hypothesis: *r*_b_ = 0) performed according to Equation (6).

The experimental protocol proposed in (32) has been extensively used in subsequent studies investigating the dynamics of protein translation from the analysis of Ribo-seq data. In this work, we are interested in estimating the ribosome drop-off rate in *S. cerevisiae* both in standard and in stressing conditions. Therefore, we selected two additional studies from the literature (namely (33) and (34)) where the Ribo-seq data were produced following the experimental protocol described in (32) and according to the experimental settings we are interested in.

In (33), Zou *et al.* studied the translational profile of *S. cerevisiae* through the analysis of Ribo-seq and RNA-seq data obtained from cells cultured in a methionine-restricted medium and is compared with the ‘control’ counterpart, cultured in standard conditions. We used all samples in this dataset, referred to as *D*1 and *D*3 (Table 1) from which we estimated the respective ribosome drop-off rates reported in Table 2.

The study presented in (34) investigates the relationships between caloric restriction and extended life span in *S. cerevisiae*. To this aim, Ribo-seq and RNA-seq data is collected from cells growing in conditions of glucose restriction and the obtained ribosome profiles are related with the corresponding reference data. The datasets we refer to as *D*2 and *D*4 in this work (Table 1), are taken from (34). We applied the Ribofilio pipeline on these datasets obtaining the results reported in Table 2).

### The Ribofilio pipeline

Ribofilio is a novel, open source software tool coded in Python that estimates the ribosome drop-off rate from the analysis of Ribo-seq data. It inputs the genome transcripts, the Ribo-seq reads (in .bed format) and if the RNA-seq data is provided, the Ribo-seq counts are normalised using the counts associated with the RNA-seq reads. To ensure the robustness of the software, extensive module testing was applied, reaching a test coverage of 81$\%$. See Supplementary Section S1.B for more details about test coverage.

To foster the usability, reproducibility and replicability of the analysis, Ribofilio is embedded into a snakemake (35) pipeline which is hosted in a public git-hub repository (https://github.com/SherineAwad/ribofilio/) where it is version controlled and extensively documented. A simple statement (‘--use-conda‘) allows us to pull any environment that was used in previous runs, thus replicating straightforwardly any computation. In addition, the implementation of the pipeline offers the possibility to perform the whole analysis workflow starting from the raw data, namely the .fastq reads resulting from the sequencing process and can be easily tailored to a broad spectrum of case studies, including the estimation of the drop-off rate on subsets of genes of interest.

The pipeline is composed of two subsequent parts, described in greater details in the following paragraphs. In the first step, the input data is pre-processed and prepared for the subsequent phase. The second step, instead, is operated by Ribofilio which produces the output of the pipeline.

#### The Ribofilio pipeline: data pre-processing

For the upstream analysis of both the Ribo-seq and the related RNA-seq data, we applied the following procedure. The raw data was filtered using trim_galore (36) (release 0.6.7) to filter out poor quality reads and remove adapters contamination. Adapters are artificial pieces of DNA introduced prior to sequencing to ensure that the DNA fragment being sequenced attaches to the sequencing flow cell which if not removed, will interfere with downstream analysis (37–39) . This refinement of the read sequences allows to reduce the probability of errors in the subsequent mapping phase; the presence of mis-sequenced nucleotides introduces artefacts that can increase the similarities between the query sequences and wrong mapping positions in the reference genome, thus increasing the probability of incorrect mapping. More specifically, we used -a parameter in trim_galore to specify adapters to be removed for each data set (personal communication, see Supplementary Table S2). We then filtered out all the reads that were shorter than 20 nucleotides to reduce the prevalence of multi-mapping errors. Shorter reads have a much higher chance of mapping to multiple places in the reference genome, simply due to combinatorics; thus with short reads, we cannot be confident that the part of the genome that the read mapped to actually reflects the origin of the read. Afterwards, the remaining reads were mapped against yeast transcripts Saccharomyces_cerevisiae.R64-1-1.cdna.all.fa to filter out the reads coming from the sequencing of rRNAs. We used the Bowtie2 aligner (40) (release 2.4.5). Finally, we generated a bed format file using the ‘bedtools bamtobed’ command (41). Supplementary Table S1 reports the primary alignments percentages for all the analysed datasets. To allow the reproducibility of the analysis we performed, we included in the Github repository a Makefile to pull from the source database the datasets and the transcripts we used.

#### The Ribofilio pipeline: computing the ribosome drop-off rate

In the second part of the pipeline, Ribofilio estimates the ribosomes drop-off rate from the analysis of the aligned Ribo-seq reads produced in the previous step.

Ribofilio implements the algorithm successfully used in (5), to which we refer the reader for the details. The specifics of the implementation we use in this work are reported in Section S1.A. In summary, we divide each reference transcript in bins of *l* nucleotides and we count the total amount of Ribo-seq reads having their 3’ ends mapping in each bin, considering all the reference features. If the RNAseq reads are available, we normalise the count of the Ribo-seq reads in each bin with the abundance of the corresponding RNA-seq reads mapping in the same bin. See Section S1.A Equation (5). Finally, we average the number of (normalised) Ribo-seq reads by dividing the total amount of reads by the number of genes that cover the bin, with an adjustment for genes that only partially cover one bin, according to (5).

The result of this process is one discrete vector *Y* that contains the (normalized) number of Ribo-seq per bin averaged over the whole set of genes considered.

We then use the vector *Y* to compute the drop-off rate per bin *r*_b_, as detailed in the next paragraphs.

### Theoretical foundations

#### Exponential distribution of ribosome density

Following the theoretical considerations expressed in (5) and and (23), we hypothesise that ribosome drop-off is a random event occurring with a probability which is independent on the position of the ribosome on the CDS and on the other ribosomes translating the same mRNA.

We consider a spatial Poisson process, with a fixed dropping rate per step. Unlike decay process in which decay rate is a constant giving the mean number of decaying particles per unit of time, in this spatial Poisson process, the rate gives the number of steps per unit length. An approximation derives from the fact that the spatial process of translation is over a discrete set of steps (the codons). If we exclude collisions between the ribosomes (this assumption is approximately true when initiation is the rate-limiting step), the run distance before drop-off is approximately exponentially distributed (more precisely, it is a geometric distribution that for large distances is very well approximated by an exponential distribution).

From this it follows that in a large population of mRNA in which initiation is asynchronous the probability density to find a ribosome at position *X* is a negative exponential. The distribution can be formulated as follows:


(1) \begin{eqnarray*} Y=Ae ^ {-r_bX} \end{eqnarray*}

where *X* is the bin number ( *X* ∈ {1, 2, …, *N*} ), *A* is the intercept (which is of no interest for us here) and *r*_b_ represents the drop-off rate *per bin*. As mentioned in the previous subsection, the variable *Y* gives the mean number of Ribos-seq reads found in position *X*, which we interpret as the expected density of ribosomes at this position at a steady state translation process in the cell population (se detailed calculations and definitions in the Supplementary Materials).

Ribofilio estimates *r*_b_ by regressing log (*Y*) on *X* through a linear regression. Indeed, if the data is distributed according to an exponential model, it can be fit by a straight line with slope *r*_b_ in a semi-logarithmic plot. More precisely, to take into account the peculiar variability of the data, Ribofilio performs a weighted linear regression, where the elements of *Y* that are supported by a greater number of genes (typically located at the beginning of the vector) have a greater weight in the regression.

The drop-off rate *per codonr*_c_, in turn, can be calculated starting from *r*_b_. Considering a bin of length (in number of codons) *l*_c_, then the probability that the ribosome does not drop-off within a bin of *l*_c_ codons is $(1 - r_c)^{l_c}$. Consequently, the probability *r*_*b*_ that any ribosome drops-off anywhere within the bin is $1 - (1 - r_c )^{l_c}$ and the drop-off rate per codon is $r_c = 1 - (1 - r_b)^{1/{l_c}}$

#### Estimate of *r*_b_ and its standard error

Given that we determine *r*_b_ by regressing experimental data, both the statistical significance of the estimated value and the associated error should be computed.

We express the error associated with *r*_b_ in terms of the confidence interval (CI), that we obtain starting from the standard error of the regression *S*_*E*_ and the margin of error *M*_*E*_ which, in turn, are computed according to Equations (2) and (3) respectively:


(2) \begin{eqnarray*} S_E = \sqrt{ \frac{\sum {(y_i -\hat{y}})^2}{N-2} }/\sqrt{\sum {(x_i -\bar{x})^2}} \end{eqnarray*}

(3) \begin{eqnarray*} M_E = t_{\alpha /2} \times S_E \end{eqnarray*}

where the critical value is calculated using a 95% confidence level (α = 0.05), according to Equation (4):


(4) \begin{eqnarray*} 1-\alpha /2 = T(t_{\alpha /2}) \end{eqnarray*}

where *T*(*t*_α/2_) is the (cumulative) distribution function of the t distribution with *N* − 2 degrees of freedom computed at *t*_α/2_. Here, *N* is the number of bins considered. Finally, from the margin of error we obtain the confidence interval:


(5) \begin{eqnarray*} CI = r_b \pm M_E. \end{eqnarray*}

To evaluate to which extent the relationship between the average number of reads *Y* and the number of bins holds (i.e. to test the null hypothesis that *r*_b_ is equal to zero), we performed a *t*-test. In particular, we determine the t-score (*t*) using Equation (6):


(6) \begin{eqnarray*} t = \frac{r_b}{S_E} \end{eqnarray*}

and we compute the *P*-value (one-sided, because the alternative hypothesis is *r*_*b*_ > 0) using the *t*-score (*t*) and *N* − 2 degrees of freedom.

To evaluate to which extent the data fits the exponential model that we use to describe the trend of the ribosome density along the CDS, we rely on two widely used metrics, namely the root mean square error (RMSE) and the coefficient of determination (*R*^2^). In this way we can evaluate the goodness of fit of our model from two different perspectives. Indeed, the *RMSE* is calculated as the standard deviation of the residuals and, thus, it evaluates the dispersion of the data around the predicted (model) values: the lower the RMSE, the more the data is distributed close to the model curve. While mathematically related to the RMSE, *R*^2^ estimates the proportion of the variance in the data that can be ‘explained’ by the predictor variable (number of bins), thus informing us in our case on how well the exponential model explains the variability of the ribosome density along the CDS. The closer *R*^2^ is to 1, the better the fit.

#### Comparing drop-off rates from different datasets

To compare the drop-off rates computed from two different datasets we used the Welch’s t-test, thus computing the t-score (*t*) following Equation (7):


(7) \begin{eqnarray*} \rm {t} = \frac{(r_{b_1} - r_{b_2}) }{\sqrt{SE_1^2 + SE_2^2}} \end{eqnarray*}

where $r_{b_1}$ and $r_{b_2}$ are the drop-off rates of group 1 and group 2 respectively and *SE*_1_ and *SE*_2_ are the standard error of group 1 and group 2 respectively. From the t-score we computed the p-value (two-sided, because the alternative hypothesis is that $r_{b_1}\ne r_{b_2}$) to evaluate the probability that the null hypothesis (the compared drop-off rate are the same) is true.

## Results

We used the Ribofilio pipeline to estimate the Ribosome drop-off rate in *S. cerevisiae* from the analysis of the Ribo-seq data listed in Table 1. This data was collected in different experimental settings, thus allowing us to compute the drop-off rate both in standard (‘control’) and non-standard conditions.

### Estimating the ribosome drop-off rate in *S. cerevisiae*

We estimated the ‘basal’ Ribosome drop-off rate from the analysis of the control datasets *D*1, *D*2. Table 2 summarises the results we obtained and highlights, in particular, the parameters (namely, RMSE and *R*^2^) that estimate the goodness of fit with the exponential model underlying ribofilio and the correspondent outcomes of the *t*-test (Equation 6) that evaluates to which extent the slope of the fitting curve (and, thus, the drop-off rate) is different from zero.

**Figure 1.**
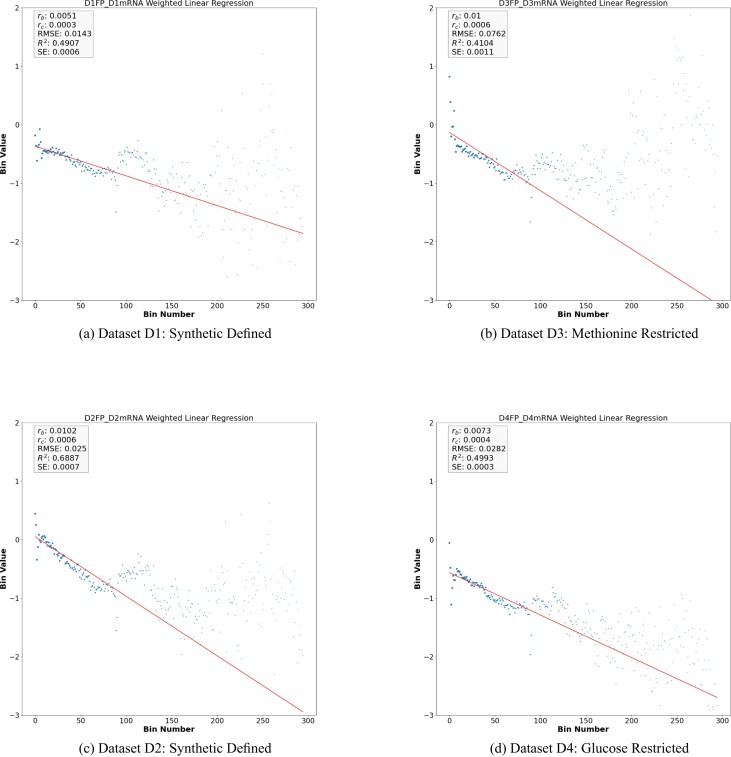
Weighted linear regression plot using bin-size equals 50 for the *Y* vector for D1, D2, D3 and D4. (see Equation 6 in supplementary material). The x-axis is the bin number and the y-axis is the *Y* vector in Log exponential. The red line corresponds to the drop-off rate *r*_*b*_.

As it is apparent by observing the log-linear plots depicted in Figure 1A and C the data coming from the analysed datasets can be fitted by a straight line having a slope *r*_*b*_ significantly different from zero (*t*-test *P*-value < 0.05 Eq. 6) which, referring to Equation (1) corresponds to the Ribosome drop-off rate per bin. Noticeably, these measurements turned out to be independent on the chosen bin size, as reported in Supplementary Table S14. Supplementary Figure S5 in the supplementary material shows *r*_*b*_ versus *r*_*c*_ rates in the four main datasets D1, D2, D3 and D4.

**Figure 2.**
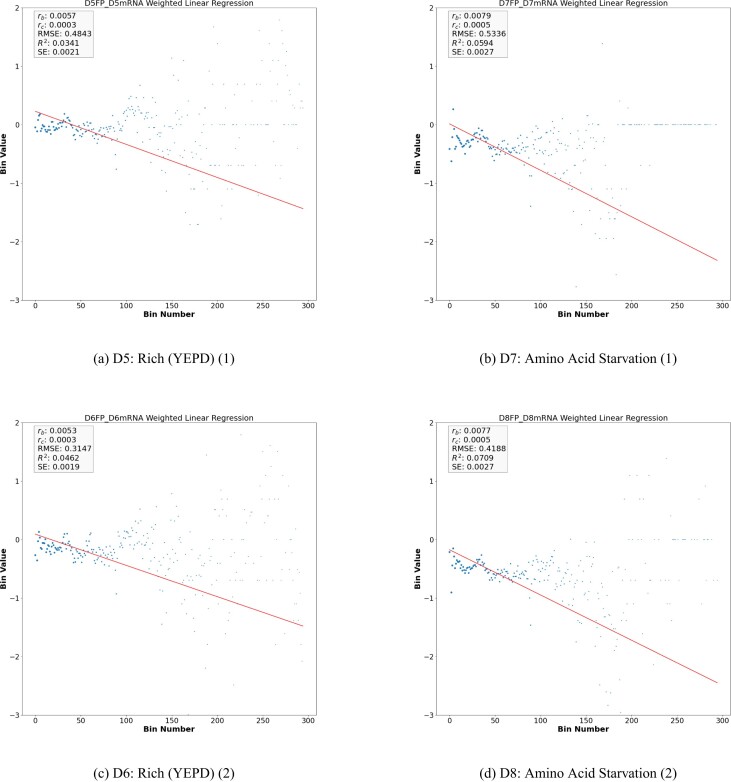
Weighted linear regression plot using bin-size equals 50 for the *Y* vector for D5, D6, D7, and D8. (see Equation 6 in supplementary material). The x-axis is the bin number and the y-axis is the *Y* vector in Log exponential. The red line corresponds to the drop-off rate *r*_*b*_.

Summing up, our analysis reveals that, in standard conditions, the average density of the ribosomes along the CDS of *S. cerevisiae* decays exponentially with a rate *r*_*c*_ per codon ranging in the order of magnitude of 10^−4^. Surprisingly, this value is consistent with the correspondent values obtained *in vitro* and *in silico* for *E. coli* (5,22). However, it is worth noticing that, differently from the case of *E. coli*, in *S. cerevisiae* the exponential model fits sub-optimally the decay of the average ribosome density along the CDS. Indeed, either the estimation of the amount of data variability explained by the model (*R*^2^) and of the goodness of fit (*RMSE*) associated with *r*_*b*_ (Table 2) suggest that a more complex scenario might underlie the dynamics of ribosome trafficking in yeast which is consistent with the greater complexity of *S. cerevisiae* with respect to *E. coli*. Interestingly, by looking either at Table 2 or Figures 1A, C, 2A and C, we notice that the choice of the growth medium could potentially affect the ribosome drop-off rate in the control case. Indeed, while *r*_*b*_ and *r*_*c*_ are not varying significantly, the parameters evaluating the goodness of fit turn out to be sensibly different depending on whether the Synthetic Defined medium or YEPD is used. This feature suggests that the growth medium might condition the protein translation’s dynamics in a subset of genes in such a way that it is not captured by estimating the drop-of rate at the whole dataset level.

Within this context, the most parsimonious hypothesis is that the global drop-off rate is determined by factors that act differently on different (group or classes of) genes and that in each (group or classes of) genes the drop off rate is constant with the length. Thus, the average distribution of the ribosome density along the CDS results from the contribution of multiple processes that, when singled out, can be described by independent exponential decay models.

Enjoying the flexibility of Ribofilio which makes it easy to focus the analysis on subsets of genes, we tested our hypothesis by computing the ribosome drop-off rate in different groups of genes, classified according functional criteria (GEO ontologies) or to structural criteria, i.e. the genes length.

Our analysis based on the GEO categories (Table 2 and Supplementary Table S4, Supplementary Table S5, and Supplementary Table S6) provided with no conclusive information about the relationship between genes’ functions and the ribosome drop-off rate. Instead, we found that *r*_*b*_ varies significantly depending on the genes length. Intriguingly, it turned out that lower drop-off rates are associated with longer genes. See Supplementary Tables S9 and S10 in the supplementary text for a significant t-test comparison of dataset D1 and D2 drop-off rate vs its corresponding drop-off rate of GO subset respectively.

### The length of the translated genes affects the ribosome drop-off rate

To identify a possible relationship between genes length and ribosome drop-off rate, we run Ribofilio using subset mode ‘-s‘ on different gene length windows, namely (]0, 500], ]500, 1000], ]1000, 2000], ]2000, 3000], ]3000, 4000], ]4000, 5000] and >5000).

**Figure 3.**
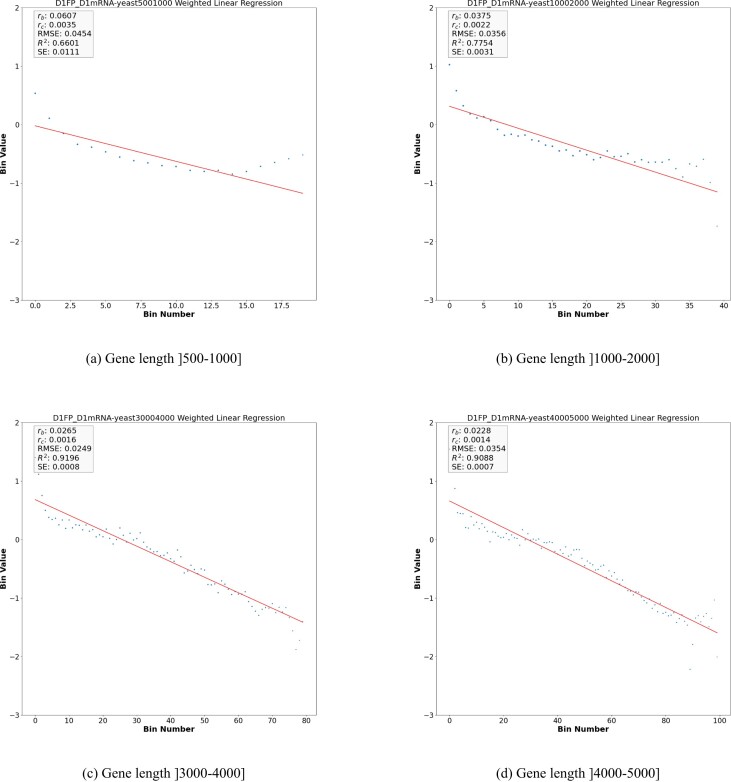
Weighted linear regression plot using bin-size equals 50 for the *Y* vector for the control dataset D1 (synthetic defined) using different gene length cutoffs. (see Equation 6 in supplementary material). The x-axis is the bin number and the y-axis is the *Y* vector in Log exponential. The red line corresponds to the drop-off rate *r*_*b*_.

The genes-length-specifc values of *r*_*b*_ and *r*_*c*_ estimated for all the datasets considered in this paper are reported in Table 3 and Supplementary Tables S7 and S8. Figure 3(A), (B), (C) and (D) illustrate a sample of the results obtained for the dataset D1. Additional plots are reported in Supplementary Figures S2, S3, and S4. A complete set of Figures can be found in the supplementary material folder hosted in the Github repository https://github.com/SherineAwad/Ribofilio/tree/master/supplementary_materials/.

**Table 3.** Drop-off rate per codon for dataset D1 and dataset D2 per Gene Length subsets

**D1**							
Gene Length	Drop-off rate per bin (*r*_*b*_)	Drop-off rate per codon (*r*_*c*_)	RMSE	$\boldsymbol{R^2}$	SE	CI	*P*-value
<500	0.0275	−0.0017	0.0628	0.0663	0.0362	*r*_*b*_ ± 0.0834	0.2341
]500–1000]	0.0607	0.0035	0.0454	0.6601	0.0111	*r*_*b*_ ± 0.0234	<0.00001
]1000–2000]	0.0375	0.0022	0.0356	0.7754	0.0031	*r*_*b*_ ± 0.0063	<0.00001
]2000–3000]	0.0281	0.0017	0.0302	0.8478	0.0013	*r*_*b*_ ± 0.0026	<0.00001
]3000–4000]	0.0265	0.0016	0.0249	0.9196	0.0008	*r*_*b*_ ± 0.0016	<0.00001
]4000–5000]	0.0228	0.0014	0.0354	0.9088	0.0007	*r*_*b*_ ± 0.0014	<0.00001
>5000	0.0133	0.0008	0.0784	0.8355	0.0007	*r*_*b*_ ± 0.0013	<0.00001
**D2**							
**Gene Length**	**Drop-off rate per bin (*r*_*b*_)**	**Drop-off rate per codon (*r*_*c*_)**	**RMSE**	** $\boldsymbol{R^2}$ **	**SE**	**CI**	**p-value**
<500	−0.0304	−0.0018	0.0586	0.0846	0.0274	*r*_*b*_ ± 0.0631	0.1499
]500–1000]	0.1104	0.0063	0.0272	0.9144	0.007	*r*_*b*_ ± 0.0147	<0.00001
]1000–2000]	0.0589	0.0034	0.0368	0.8914	0.0032	*r*_*b*_ ± 0.0065	<0.00001
]2000–3000]	0.0378	0.0022	0.0402	0.8831	0.0016	*r*_*b*_ ± 0.0031	<0.00001
]3000–4000]	0.0328	0.0019	0.0436	0.9089	0.0011	*r*_*b*_ ± 0.0023	<0.00001
]4000–5000]	0.0268	0.0016	0.0477	0.9113	0.0009	*r*_*b*_ ± 0.0017	<0.00001
>5000	0.0139	0.0008	0.0818	0.8407	0.0006	*r*_*b*_ ± 0.0012	<0.00001

Column 1: Gene length sub-group. Column 2: Drop-off rate per bin (rb). Column 3: Drop-off rate per codon (rc). Column 4: RMSE. Column 5: coefficient of determination (R2). Column 6: Standard error estimate (SE). Column 7: Confidence interval 95%. Column 8: *P*-value resulting from the *t*-test (null hypothesis: *r*_b_ = 0) performed according to Equation (6).

**Figure 4.**
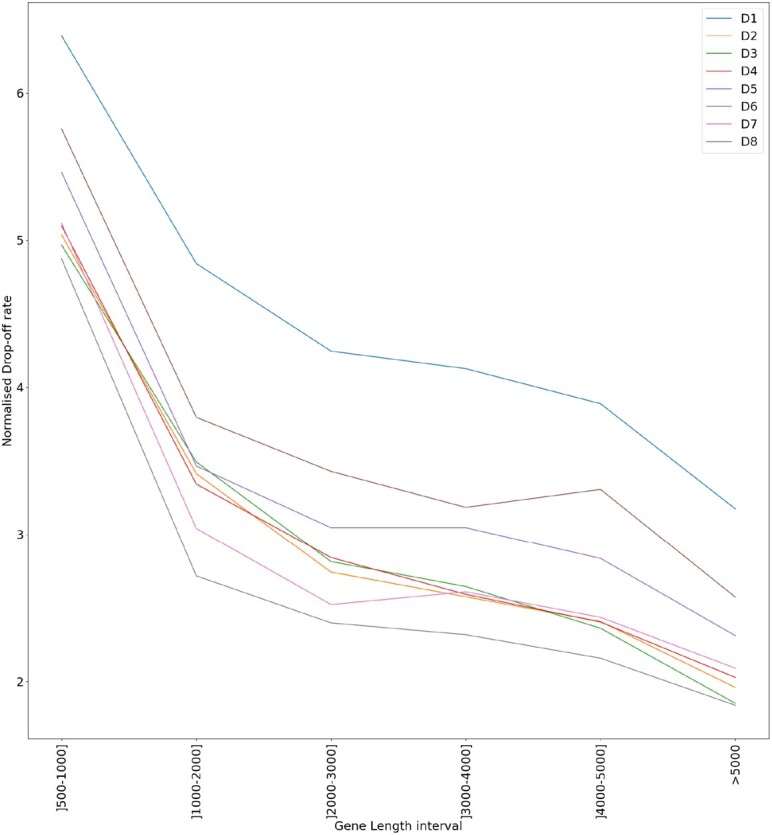
Drop-off rate per bin (*r*_*b*_) for each gene length subset group. The different colours represent the different analysed datasets. The values of *r*_*b*_ are standardised to facilitate the visualisation. The subset [0–500] is removed from this plot because the associated values of *r*_*b*_ turned out to be not significantly different from zero.

By inspecting the results we notice that, excluding the case of the shortest genes (gene length < 500), all the obtained drop-off rates turned out to be significantly different from zero (*P*-value < 0.001) and also significantly different from the correspondent values computed when all the genes are considered independently on their lengths (Supplementary Tables S11, S12 and S13). Moreover, both *R*^2^ and the RMSE turned out to be improved with respect to the correspondent values associated with the global values of *r*_*b*_, thus supporting our hypothesis that the ribosome density along the CDS decays exponentially with different rates depending on the gene length. In this scenario, the ribosome drop-off rate estimated at the global level would result from the interplay between the length-specific rates. Figure 4 summarises this result. See Supplementary Figures S1 for genes length distribution of *S. cerevisiae*.

The absence of a detectable drop-off rate for short genes (length ≤ 500) remains puzzling and, in general, calls for a more finely tuned ribosomes processivity in *S. cerevisiae* when compared to *E. coli*.

### Variability of the (gene length-specific) ribosome drop-off rate with respect to the environmental conditions

To examine environmental factors that could affect the ribosome drop-off rate, possibly in a gene-specific way, we extended our analysis to the data collected in experiments where *S. cerevisiae* was cultured in stressing environmental conditions. In particular, we considered the datasets *D*3, *D*4 (*S. cerevisiae* grown in methionine and glucose restricted media, respectively) *D*7 and *D*8, referring to acute amino-acid starvation and we compared them with the corresponding control datasets, namely *D*1, *D*2, *D*5 and *D*6 respectively.

First, we checked whether the different experimental conditions might influence the drop-off rate at the global level. Table 4 reports the results of the Welch’s *t*-test (Equation 7) that we used to evaluate possible differences between the results obtained in the different experimental conditions and the respective reference counterparts. See Supplementary Table S17 in the supplementary text for the gene length subsets sizes.

**Table 4.** Welch’s *t*-test to compare ‘control’ and test of D1 versus D3, D2 versus D4, D5 versus D7 and D6 versus D8

Dataset ID	*P*-value
D1 versus D3	0.0001
D2 versus D4	0.0001
D5 versus D7	0.2602
D6 versus D8	0.2338

Column 1: Dataset ID (Table 1 for the respective GEO coordinates). Column 2: *P*-value.

Inspecting Table 4 we notice that only in two out of four cases (methionine and glucose restriction) the drop-off rate was significantly different from the reference dataset, albeit the direction of the variation (greater and smaller than the reference, respectively, Table 2) is towards opposite directions.

We studied the drop-off rate of treatment datasets (D3 and D4) of the control datasets (D1 and D2) respectively. See Figure 1B and D, respectively and see Table 2 for the regression fits. To examine whether the treatment condition changes the drop-off rate, we compared the drop-off rate of the control dataset and its corresponding treatment dataset. Table 2 shows the drop-off rate and the corresponding fitting statistics of treatment datasets D3 and D4 when compared to the control datasets D1 and D2, respectively.

We also examined the drop-off rate under both rich and starvation conditions. Table 2 shows the drop-off rate for under rich conditions (datasets D5 and D6), and under starvation conditions (datasets D7 and D8. See Figure 2A and C and B and D, respectively). Table 2 shows there is no significant change in drop-off rate when comparing rich conditions (D5 and D6) with starvation conditions (D7 and D8). Although there is a significant change of the drop-off rate under the treatment condition (see Table 2), the low RMSE and *R*^2^ show that there are other factors affecting the drop-off rate rather than the rich and starvation conditions.

### Variability of the ribosome drop-off rate with respect to the functional (gene ontology) category

To further investigate other factors that possibly affect the drop-off rate, we run ‘ribofilio‘ using subset mode ‘-s‘ on different gene ontology terms. See Supplementary Table S3 for the details of each GO category description and set size. Supplementary Tables S4-S6 show the drop-off rate and its corresponding regression fitting statistics for each GO term per each control dataset. Response to stress (GO:0006950) and Transmembrane Transporter Activity (GO:0022857) show significant fitting statistics (*P-*value < 0.01) when compared to control datasets D1 and D2. However, other gene ontology categories show random or no significance across control datasets which deems the results inconclusive about the relationship between gene ontology and drop-off rate.

## Discussion and conclusion

An accurate estimation of the ribosome drop-off rate turns out to be fundamental in a broad spectrum of cases where the mRNA translation efficiency needs to be determined. Ribofilio is a novel piece of software produced to compute the ribosome drop-off rate when Ribo-seq data (and, optionally, the RNAseq data) is available. We offer Ribofilio embedded into a module-tested and FAIR pipeline, made available as an open-source tool and maintained (version controlled) in a dedicated GitHub repository. Remarkably, the Ribofilio pipeline is designed to be run also on subsets of genes, grouped according to the user’s interest. Section S4, for instance, presents a case study where Ribofilio is run on single genes, namely MSH2 and MLH1. Supplementary Tables S15 and S16 show the drop-off rate of the control data sets D1 and D2 and the treatment dataset D3, D4 respectively on genes MSH2 and MLH1.

We used the Ribofilio pipeline on Ribo-seq data produced for *S. cerevisiae* and we found that ribosome drop-off events occur at a significant rate that we estimated in various experimental conditions.

The algorithm implemented by Ribofilio, based on (5), assumes a position-independent, constant probability of ribosome drop-off which entails a negative exponential distribution of the ribosomes density along the Coding Sequence (CDS). In (5) it was shown that this model provides an accurate description of the average trend of the ribosomes density along the *E. coli*’s Open Reading Frames. In this work, we determined that also in *S. cerevisiae* the ribosomes density, on average, decays exponentially along the CDS. Noticeably, in this case, we observed a more ‘noisy’ trend of the data around the regression curve as indicated by the value of the parameters that estimates the goodness of fit.

It’s worth noticing that the deviation from the ‘straight line’ at larger length scales is due to the lack of statistics, since very few genes contribute to this part of the data and therefore the individual, gene-to-gene variations dominate the behaviour of the mean coverage. At short length scales, close to the start codon, there is an accumulation of reads commonly believed to be the so-called ‘ramp’, a region of the ORF in which traffic jam seem to occur and that therefore does not fulfill the conditions for the exponential decay . More in general, a possible interpretation of the observed sub-optimal fit of the exponential model is that when all the genes of *S. cerevisiae* are considered together, the trend of the ribosomes density along the CDS results from the concurrent interplay of multiple factors acting differently at the single gene’s level. In other words, the sub-optimal fit of the exponential model shown, e.g., in Figure 1 suggests that either gene-specific features or environmental conditions could affect the ribosomal drop-off rate.

To gain possible insights on the impact of environmental conditions, we considered the drop-off rate computed in datasets exposed to different environmental conditions such as, e.g. datasets D5-D8. Despite we observed some differences in the goodness of fit depending on the growth medium, we did not found any measurable evidence correlating the ribosome drop-off rate with the tested environmental conditions.

To study the impact of the gene-specific features on the overall dynamics, we computed the ribosome drop-off rates in subsets of genes partitioned according to different criteria, such as their functional role (based on the GO classification) or the length of the respective CDS. Our results show that the genes’ functional role is not a factor that affect the drop-off rate but that the genes’ length could be. More precisely, our results show that genes (CDS) with different lengths exhibit significantly different ribosome drop-off rates. Therefore, when all the genes are considered together independently on their length, the overall drop-off rate results from the combination of the single length dependent rates.

Interestingly, we also noticed that longer CDS are associated with lower drop-off rates. This relationship between the measured drop-off rate and the length of the CDS entails relevant consequences at the biological level.

### Biological relevance of the ribosome drop-off rate

In general, when *S. cerevisiae* is cultured in standard ‘control’ conditions, we determined that the order of magnitude of the ribosome drop-off rate is in the range of 10^−4^ per codon.

Albeit it could look negligible, this value becomes in fact relevant when it is evaluated in the context of the protein translation process at the molecular level.

To gain a better insight of the impact of ribosome drop-off in this context, it is useful to compute the probability *P*_*S*_ for a ribosome to reach the stop codon of the CDS, thus terminating the synthesis of a full protein. The value of *P*_*S*_ can be computed directly from the estimated *r*_*c*_. Indeed, given that according to our model the ribosome density decays exponentially along the CDS with rate *r*_*c*_, the probability that a ribosome will reach the stop codon located *L* codons far from the start is: $P_s = (1-r_c)^L \sim e^{-r_cL}$ when *r*_*b*_ ≪ 1.

As an illustrative example, if we consider a CDS of the length of 100 codons (which stays within the first quartile of *S. cereveisiae*’s CDS length distribution) and *r*_*c*_ = 10^−4^ , *P*_*S*_ assumes a value of 0.99 which means that, on average, about $99\%$ of the ribosomes that initiated the translation process will reach the stop codon. If, instead, if we consider a CDS of the one of the longest CDS length of S. cerevisiae, namely YLR106C with *L* = 4911 *P*_*S*_ would be 0.611 which means that only $61.1\%$ of the ribosomes would reach the 3′ end of the CDS.

This example suggests that if the ribosome drop-off rate was the same for all the genes (and equal to the rate measured globally), longer genes would not be translated reliably.

Interestingly, our results show that the ribosome drop-off rate is lower in longer CDSs, thus suggesting the possible existence of ‘compensatory features’ in longer CDSs that leads to the reduction of the drop-off rate.

Once ribosomes terminate (either prematurely or at the stop codon) they can be recycled. This drop-off rate concerns the unspecific premature stop of translation by the ribosomes which represents a waste for the cell. It may also represent a resource to make sure that ribosomes stop translation of unnecessary proteins in cases of acute stress. In addition, the drop-off rate affects longer genes. The translation of longer genes is less efficient because the probability to successfully reach the stop codon decreases exponentially with the length of the mRNA.

Despite ribosomes could drop-off with a lower probability when translating longer mRNAs, our measurements would suggest that the efficiency of the translation can be related to the length of the translated CDS, with less efficiency associated with longer transcripts, due to the greater number (on average) of drop-off events. This observation is consistent with the well known experimental evidence showing that, in a broad range of organisms including S. cerevisiae, the average ribosomal density is inversely correlated with the CDS’s length (26).

Our model assumes at first that initiation rate is the rate limiting step. While we realised that this was consistent as well in our results in *E. coli*, it may be not true for all genes and not true for all organisms. In fact, we are interested only in the probability to drop-off. Once the drop-off probability is known, the probability to reach the end of the mRNA is distributed according to a geometric distribution, which resembles an exponential distribution in the continuous approximation. In addition, the drop-off rate presented in this paper is not estimated at the level of single sequences. The reason for this is that the data is too noisy to determine a trend. We also think that the finding of the drop-off variation as a function of the length of the genes is puzzling and deserves the role of becoming an hypothesis to be tested experimentally with specific tools.

## Supplementary Material

lqae036_Supplemental_File

## Data Availability

All the data used in the paper is publicly available in the GEO data repository. The interested users might wish to use the following Makefile to pull this data automatically: https://github.com/SherineAwad/Ribofilio/blob/master/data/Makefile and https://doi.org/10.17617/3.H7OPIB.
