# Supplementary material for: A data-driven estimation of the ribosome drop-off rate in S. cerevisiae reveals a correlation with the genes length
Source: NAR Genom Bioinform. 2024 Apr 18;6(2):lqae036. doi: 10.1093/nargab/lqae036 (PMC11025885; doi:10.1093/nargab/lqae036)
Supplement: lqae036_Supplemental_File [file lqae036_supplemental_file.pdf]

Published online *Nucleic Acids Research, 0000, Vol. 00, No. 00* ***1–19***
doi:10.1093/nar/gkn000

Supplementary materials
A data-driven estimation of the ribosome drop-off rate in *S. cerevisiae* reveals a correlation with the genes length.

Sherine Awad, Angelo Valleriani, Davide Chiarugi

© The Author(s)
This is an Open Access article distributed under the terms of the Creative Commons Attribution Non-Commercial License (http://creativecommons.org/licenses/by-nc/2.0/uk/) which permits unrestricted non-commercial use, distribution, and reproduction in any medium, provided the original work is properly cited.

S1.A COMPUTING THE AVERAGE NUMBER OF RPFS PER ORF: A BINNING STRATEGY

Here, we explain the core of 'ribofilio'. For each dataset reported in Table 1, we created a nucleotides vector positions as follows. For any gene g, let $\ell(g)$ be the length of the ORF in nucleotides. We first create a vector of the length equal to the longest ORF, in nucleotides. At each position i of this vector, we count how many ORF have a length larger or equal to i. This gives the vector

$$GeneCoverage_i = \sum_g \mathrm{bool}(\ell(g) \geq i) \quad (1)$$

where the sum runs over all genes and the function bool is defined as

$$\mathrm{bool}(x) = \begin{cases} 1 \text{ if } x = \mathrm{True} \\ 0 \text{ if } x = \mathrm{False}. \end{cases} \quad (2)$$

As a next step, we build a vector that contains information about the 3' position of all reads collected in the sample. Let r be any read in the sample, then the function $t(r)$ gives the position i on the corresponding ORF of the 3' end of the read r. In analogy to what done above we build now a vector of the same length as the longest ORF. This vector contains in each element i the number of reads in the entire sample whose 3' end falls exactly on i. This could be formally defined as

$$nucleotidesMatrix_i = \sum_r \mathrm{bool}(t(r) = i) \quad (3)$$

where the sum runs over all ribo-Seq reads of the sample. Finally, the quantity of interest is the vector

$$positions_i = \frac{nucleotidesMatrix_i}{GeneCoverage_i}. \quad (4)$$

To start binning, we group all normalised positions into bins of size bin-size. We use bins of size l to sum up the elements of the vector $position_i$ into our BIN vector. This results in the BIN vector as in equation **5**):

$$BIN_{(j)} = \frac{1}{l} \sum_{i=1+l(j-1)}^{jl} positions_i \quad (5)$$

where l is the bin-size. We use the RPF reads to generate the BIN vector using equation **5** and similarly use their corresponding mRNA reads to generate the BIN vector. To normalize the amount of RPFs with the abundance of the corresponding mRNA reads, we divided the value in each cell of the footprints BIN vector by their corresponding cell of mRNA BIN as in equation **6**

$$Y_{(i)} = \frac{\text{Footprints } Bin_i}{\text{mRNA } Bin_i} \quad (6)$$

Where Y is the normalised RPF vector that we fit in Eq. (1) of the main text.

A weighted linear regression is then performed where we regress over the BIN vector and the weight is basically the gene coverage per bin, i.e how many genes could possibly cover a given bin according to the gene's length.

So basically, the gene coverage at a position is actually how many genes can cover this position based on the gene length. Once we count how many ribosomes reads cover each position from ribo-seq reads, we normalise this ribosomes reads count with the gene coverage at each position. Then we sums up the ribosomes reads normalised counts in each bin. A weighted linear regression is performed where the weight is the gene coverage per bin. i.e how many genes can cover this bin based on the genes

length. This way the variability of the coding sequence is considered in normalising the ribosomes reads counts and weights of regression are assigned accordingly.

S1.B TEST COVERAGE

Test coverage is defined as a metric in Software Testing that measures the amount of testing performed by a set of tests. It will include gathering information about which parts of a program are executed when running the test suite to determine which branches of conditional statements have been taken. Test coverage should be 70% or more for a good tested software module (4)
.

S2. SUPPLEMENTARY TABLES

Table S1. Primary alignments percentages for each dataset. See main text Table 1 for the respective GEO coordinates

Dataset	FP %	mRNA %
D1	32.98	41.07
D2	43.90	39.53
D3	28.63	39.52
D4	42.16	41.09
D5	73.79	47.00
D6	40.83	46.22
D7	64.40	41.50
D8	48.79	32.45

Table S2. Adapters trimmed used for each dataset as confirmed by authors. See main text Table 1 for the respective GEO coordinates

Adapter	Datasets
CTGTAGGCACCATCAAT	D1, D2, D3, D4
PolyA	D5, D6, D7, D8

Table S3. Gene Ontology Description. Column 1 is Gene Ontology ID. Column 2 is Gene Ontology Description. Column 3 is set size.

Gene Ontology ID	Gene Ontology Description	Set Size
GO:0000462	Maturation of SSU-rRNA from tricistronic rRNA transcript	70
GO:0000466	Maturation of 5.8S rRNA from tricistronic rRNA transcript	15
GO:0002181	Cytoplasmic Translation	151
GO:0003723	RNA binding	315
GO:0005840	Ribosome	171
GO:0006364	rRNA processing	111
GO:0006396	RNA processing	31
GO:0006406	mRNA Export from Nucleus	36
GO:0006412	Translation	186
GO:0006950	Response to Stress	33
GO:0007049	Cell Cycle	91
GO:0009651	Response to Salt Stress	20
GO:0015934	Large Ribosomal Subunit	18
GO:0016458	Gene Silencing	2
GO:0022625	Cytosolic Large Ribosomal Subunit	83
GO:0022626	Cytosolic Ribosome	12
GO:0022627	Cytosolic Small Ribosomal Subunit	66
GO:0022857	Transmembrane Transporter Activity	111
GO:0030490	Maturation of SSU-rRNA	15
GO:0030687	Preribosome, Large Subunit Precursor	52
GO:0042254	Ribosome Biogenesis	64
GO:0042274	Ribosomal Small Subunit Biogenesis	30
GO:0042255	Ribosome Assembly	4
GO:0003735	Structural Constituent of Ribosome	220
GO:0003743	Translation Initiation Factor activity	37
GO:0030684	Preribosome	2
GO:0044249	Cellular biosynthetic process	6
GO:0006457	Protein folding	85
GO:0030686	90S Preribosome	22
GO:0008135	Translation factor activity, RNA binding	1
GO:0030529	IntracellularRibonucleoprotein complex	6
GO:0015935	Small Ribosomal subunit	18

Table S4. Drop-off rate per codon for dataset D1 per GO subsets: Column 1: Gene Ontology ID (see Supplementary Table 1 for the respective GO IDs). Column 2: Drop-off rate (r_b). Column 3: Drop-off rate per codon (r_c). Column 4: $RMSE$. Column 5: coefficient of determination (R^2). Column 6: Standard Error Estimate (SE). Column 7: Confidence Interval 95%. Column 8: p-value resulting from the t-test (null hypothesis: $r_b = 0$) performed according to Equation 6.

D1							
GO	Drop-off (r_b)	Dropoff per codon (r_c)	RMSE	R^2	SE	CI	Pvalue
GO:0000462	0.0136	0.0008	0.1568	0.301	0.0016	r_b±0.0031	<0.00001
GO:0000466	0.0114	0.0007	0.447	0.137	0.0039	r_b±0.0076	0.0019
GO:0002181	0.0139	0.0008	0.4688	0.1139	0.0057	r_b±0.0113	0.0082
GO:0003723	0.0142	0.0008	0.1771	0.2837	0.0052	r_b±0.0102	0.0034
GO:0005840	0.0023	0.0001	0.1523	0.0037	0.0041	r_b±0.0081	0.2852
GO:0006364	0.0033	0.0002	0.426	0.051	.0013	r_b±0.0026	0.0077
GO:0006396	0.0025	0.0002	0.2275	0.0137	0.0015	r_b±0.0029	0.0448
GO:0006406	0.0153	0.0009	0.2653	0.2429	0.0049	r_b±0.0098	0.0012
GO:0006412	0.029	0.0017	0.4762	0.2228	0.01	r_b±0.0198	0.0023
GO:0007049	0.0193	0.0011	0.2507	0.378	0.0046	r_b±0.0092	<0.00001
GO:0009651	0.0191	0.0011	0.1238	0.4797	0.0026	r_b±0.0052	<0.00001
GO:0015934	0.04	0.0024	3.1911	0.238	0.0115	r_b±0.0228	0.0004
GO:0016458	0.0352	0.0021	1.4971	0.3414	0.0056	r_b±0.0111	<0.00001
GO:0022625	0.0359	0.0021	0.0879	0.3203	0.0059	r_b±0.012	<0.00001
GO:0022626	0.0077	0.0005	0.1348	0.4062	0.0008	r_b±0.0016	<0.00001
GO:0022627	0.0021	0.0001	0.1957	0.0026	0.0038	r_b±0.0075	0.2915
GO:0030490	0.0031	0.0002	0.2414	0.0223	0.002	r_b±0.0041	0.0671
GO:0030687	0.0327	0.0019	0.1473	0.5615	0.0032	r_b±0.0064	<0.00001
GO:0042254	0.0122	0.0007	0.3784	0.2818	0.0025	r_b±0.005	<0.00001
GO:0042274	0.014	0.0008	0.2632	0.187	0.0065	r_b±0.013	0.0171
GO:0042255	0.0048	0.0003	0.2121	0.0371	0.0031	r_b±0.0062	0.0638
GO:0003735	0.0465	0.0027	0.1409	0.3187	0.03	r_b±0.0609	0.0653
GO:0003743	0.0079	0.0005	0.0632	0.1874	0.0018	r_b±0.0036	<0.00001
GO:0006457	0.0143	0.0008	0.0765	0.2795	0.0035	r_b±0.007	0.0001
GO:0008135	0.0728	0.0042	0.6495	0.2302	0.0305	r_b±0.0639	0.0139
GO:0030529	0.0501	0.0029	1.0464	0.1873	0.0277	r_b±0.0561	0.0394
GO:0030684	0.0406	0.0024	0.0796	0.5521	0.0068	r_b±0.0141	<0.00001
GO:0044249	0.0481	0.0028	0.0946	0.5671	0.0093	r_b±0.019	<0.00001
GO:0030686	0.0152	0.0009	0.4494	0.3564	0.0022	r_b±0.0043	<0.00001
GO:0015935	0.0391	0.0023	3.4594	0.2245	0.0116	r_b±0.023	0.0005

Table S5. Drop-off rate per codon for dataset D2 per GO subsets: Column 1: Gene Ontology ID (see Supplementary Table 1 for the respective GO IDs). Column 2: Drop-off rate (r_b). Column 3: Drop-off rate per codon (r_c). Column 4: $RMSE$. Column 5: coefficient of determination (R^2). Column 6: Standard Error Estimate (SE). Column 7: Confidence Interval 95%. Column 8: p-value resulting from the t-test (null hypothesis: $r_b = 0$) performed according to Equation 6.

D2							
GO	Drop-off (r_b)	Dropoff per codon (r_c)	RMSE	R^2.	SE	CI	Pvalue
GO:0000462	0.0236	0.0014	0.1954	0.5088	0.0017	r_b±0.0033	<0.00001
GO:0000466	0.0216	0.0013	0.2354	0.5198	0.0018	r_b±0.0035	<0.00001
GO:0002181	0.028	0.0017	0.3795	0.3932	0.0047	r_b±0.0094	<0.00001
GO:0003723	0.0226	0.0013	0.1816	0.4957	0.004	r_b±0.0079	<0.00001
GO:0005840	0.011	0.0007	0.2144	0.0556	0.0038	r_b±0.0077	0.0028
GO:0006364	0.0052	0.0003	0.308	0.1571	0.0009	r_b±0.0017	<0.00001
GO:0006396	0.0056	0.0003	0.3948	0.0379	0.0021	r_b±0.0042	0.0046
GO:0006406	0.0306	0.0018	0.0737	0.822	0.0024	r_b± 0.0047	<0.00001
GO:0006412	0.0351	0.0021	0.3148	0.3879	0.006	r_b±0.0119	<0.00001
GO:0007049	0.0289	0.0017	0.2014	0.6291	0.0022	r_b±0.0044	<0.00001
GO:0015934	0.0348	0.0021	1.0647	0.4149	0.006	r_b±0.0119	<0.00001
GO:0016458	0.0434	0.0026	1.0898	0.5196	0.005	r_b±0.0099	<0.00001
GO:0022625	0.0702	0.0041	0.1393	0.5316	0.0086	r_b±0.0175	<0.00001
GO:0022626	0.0102	0.0006	0.2022	0.4473	0.0009	r_b±0.0018	<0.00001
GO:0022627	0.015	0.0009	0.2709	0.0887	0.0047	r_b±0.0094	0.0011
GO:0030490	0.0014	0.0001	0.2782	0.004	0.0022	r_b±0.0044	0.2663
GO:0030687	0.0345	0.002	0.1269	0.6235	0.0024	r_b±0.0048	<0.00001
GO:0042254	0.0163	0.001	0.185	0.5863	0.001	r_b±0.002	<0.00001
GO:0042274	0.0215	0.0013	0.1411	0.5017	0.0023	r_b±0.0045	<0.00001
GO:0042255	0.0008	0.0	0.3551	0.0006	0.0044	r_b±0.0088	0.4283
GO:0003735	0.0807	0.0047	0.1117	0.6404	0.0074	r_b±0.015	<0.00001
GO:0003743	0.023	0.0014	0.0908	0.5782	0.002	r_b±0.004	<0.00001
GO:0006457	0.0298	0.0018	0.1517	0.46	0.0088	r_b±0.0176	0.0006
GO:0008135	0.0985	0.0057	0.7906	0.3105	0.0337	r_b±0.0705	0.0043
GO:0030529	0.0806	0.0047	1.0568	0.3713	0.0282	r_b±0.0572	0.0035
GO:0030686	0.0131	0.0008	0.2096	0.4696	0.001	r_b±0.0021	<0.00001
GO:0015935	0.0355	0.0021	1.0798	0.4343	0.006	r_b±0.012	<0.00001

Table S6. Drop-off rate per codon for dataset D1 and D2 per GO subsets. bin-size equals 50. Column 1: GO ID (Table 1 in section S2 for the respective GO names). Column 2: Set Size. Column 3: Description. Column 4: Drop-off rate (r_b). Column 5: Drop-off rate per codon (r_c). Column 6: $RMSE$. Column 7: coefficient of determination (R^2). Column 8: Standard Error Estimate (SE). Column 9: Confidence Interval 95%. Column 10: p-value resulting from the t-test (null hypothesis: $r_b = 0$) performed according to Equation 6. The response to stress GO (GO:0006950) and The Transmembrane Transporter Activity GO (GO:0006950) are commonly significant among the control datasets D1, and D2, both have p-value <0.01. Their set size is 33 and 111 respectively, and they are significantly different when compared to control datasets D1 and D2. See table S9 and S10.

D1									
GO	Size	Description	Drop-off (r_b)	drop-off per codon (r_c)	RMSE	R^2	SE	CI	p-value
**GO:0006950**	33	Response to Stress	0.2064	0.0113	3.2499	0.7287	0.021	r_b±0.0418	<0.00001
**GO:0022857**	111	Transmembrane Transporter Activity	0.04	0.0024	0.0601	0.7682	0.0042	r_b±0.0084	<0.00001
D2									
**GO:0006950**	33	Response to Stress	0.2166	0.0118	4.4808	0.6821	0.0241	r_b±0.0482	<0.00001
**GO:0022857**	111	Transmembrane Transporter Activity	0.0568	0.0033	0.0888	0.8188	0.009	r_b±0.018	<0.00001
GO:0030684	2	Preribosome	0.0594	0.0035	0.0909	0.6985	0.0078	r_b±0.016	<0.00001
GO:0044249	6	Cellular biosynthetic process	0.0747	0.0043	0.0864	0.7758	0.008	r_b± 0.0164	<0.00001
GO:0009651	20	Response to Salt Stress	0.0467	0.0027	0.2279	0.7495	0.0035	r_b±0.0069	<0.00001

Table S7. Drop-off rate for treatment dataset D3, and D4 per Gene Length subset: Column 1: Gene Length subset. Column 2: Drop-off rate (r_b). Column 3: Drop-off rate per codon (r_c). Column 4: $RMSE$. Column 5: coefficient of determination (R^2). Column 6: Standard Error Estimate (SE). Column 7: Confidence Interval 95%. Column 8: p-value resulting from the t-test (null hypothesis: $r_b = 0$) performed according to Equation 6.

D3							
Gene Length	Drop-off (r_b)	drop-off per codon (r_c)	RMSE	R^2	SE	CI	Pvalue
<500	-0.0603	-0.0035	0.0827	0.2052	0.0366	$r_b \pm 0.0843$	0.069
]500-1000]	0.1074	0.0061	0.1829	0.6011	0.019	$r_b \pm 0.04$	<0.00001
]1000-2000]	0.0596	0.0035	0.1475	0.6772	0.0055	$r_b \pm 0.0111$	<0.00001
]2000-3000]	0.0399	0.0023	0.1444	0.7009	0.0028	$r_b \pm 0.0056$	<0.00001
]3000-4000]	0.0341	0.002	0.109	0.8121	0.0017	$r_b \pm 0.0033$	<0.00001
]4000-5000]	0.0258	0.0015	0.1255	0.7834	0.0012	$r_b \pm 0.0024$	<0.00001
>5000	0.0105	0.0006	0.1508	0.6228	0.0008	$r_b \pm 0.0015$	<0.00001

D4							
Gene Length	Drop-off (r_b)	drop-off per codon (r_c)	RMSE	R^2	SE	CI	Pvalue
<500	-0.009	-0.0005	0.0652	0.0073	0.0292	$r_b \pm 0.0674$	0.3825
]500-1000]	0.0987	0.0057	0.0501	0.823	0.0091	$r_b \pm 0.0192$	<0.00001
]1000-2000]	0.0494	0.0029	0.06	0.7799	0.0039	$r_b \pm 0.0079$	<0.00001
]2000-3000]	0.0359	0.0021	0.0503	0.8454	0.0016	$r_b \pm 0.0032$	<0.00001
]3000-4000]	0.0291	0.0017	0.0429	0.8885	0.001	$r_b \pm 0.002$	<0.00001
]4000-5000]	0.0234	0.0014	0.0534	0.8748	0.0009	$r_b \pm 0.0017$	<0.00001
>5000	0.0131	0.0008	0.0573	0.8705	0.0004	$r_b \pm 0.0008$	<0.00001

Table S8. Drop-off rate for rich and starved datasets D5, D6, D7, and D8 per Gene Length subset: Column 1: Gene Length subset. Column 2: Drop-off rate (r_b). Column 3: Drop-off rate per codon (r_c). Column 4: $RMSE$. Column 5: coefficient of determination (R^2). Column 6: Standard Error Estimate (SE). Column 7: Confidence Interval 95%. Column 8: p-value resulting from the t-test (null hypothesis: $r_b = 0$) performed according to Equation 6.

D5							
Gene Length	Drop-off (r_b)	drop-off per codon (r_c)	RMSE	R^2	SE	CI	Pvalue
<500	-0.1931	-0.0107	1.9229	0.1024	0.1462	$r_b \pm 0.3371$	0.1115
]500-1000]	0.0626	0.0036	0.0167	0.8488	0.0085	$r_b \pm 0.0179$	<0.00001
]1000-2000]	0.029	0.0017	0.05	0.5943	0.0037	$r_b \pm 0.0074$	<0.00001
]2000-3000]	0.0219	0.0013	0.0389	0.7242	0.0016	$r_b \pm 0.0031$	<0.00001
]3000-4000]	0.0211	0.0013	0.0289	0.8612	0.0009	$r_b \pm 0.0019$	<0.00001
]4000-5000]	0.0189	0.0011	0.0412	0.8546	0.0008	$r_b \pm 0.0016$	<0.00001
>5000	0.0094	0.0006	0.5654	0.2604	0.0021	$r_b \pm 0.0041$	<0.00001
D6							
Gene Length	Drop-off (r_b)	drop-off per codon (r_c)	RMSE	R^2	SE	CI	Pvalue
<500	-0.117	-0.0067	1.4885	0.0513	0.1285	$r_b \pm 0.2964$	0.1945
]500-1000]	0.055	0.0032	0.0186	0.7953	0.0079	$r_b \pm 0.0166$	<0.00001
]1000-2000]	0.0278	0.0016	0.0478	0.5857	0.004	$r_b \pm 0.0082$	<0.00001
]2000-3000]	0.0212	0.0013	0.0349	0.734	0.0016	$r_b \pm 0.0032$	<0.00001
]3000-4000]	0.0187	0.0011	0.0456	0.7557	0.0012	$r_b \pm 0.0024$	<0.00001
]4000-5000]	0.0199	0.0012	0.0304	0.8985	0.0007	$r_b \pm 0.0014$	<0.00001
>5000	0.0098	0.0006	0.4446	0.328	0.0019	$r_b \pm 0.0038$	<0.00001
D7							
Gene Length	Drop-off (r_b)	drop-off per codon (r_c)	RMSE	R^2	SE	CI	Pvalue
<500	-0.1713	-0.0095	2.2961	0.0699	0.1592	$r_b \pm 0.3672$	0.1568
]500-1000]	0.0729	0.0042	0.0141	0.9004	0.0066	$r_b \pm 0.014$	<0.00001
]1000-2000]	0.0301	0.0018	0.0613	0.5638	0.0045	$r_b \pm 0.0092$	<0.00001
]2000-3000]	0.0208	0.0012	0.0575	0.6152	0.0018	$r_b \pm 0.0036$	<0.00001
]3000-4000]	0.0212	0.0013	0.0546	0.7683	0.0013	$r_b \pm 0.0026$	<0.00001
]4000-5000]	0.0181	0.0011	0.0828	0.7303	0.0011	$r_b \pm 0.0022$	<0.00001
>5000	0.0119	0.0007	1.0522	0.2324	0.0027	$r_b \pm 0.0053$	<0.00001
D8							
Gene Length	Drop-off (r_b)	drop-off per codon (r_c)	RMSE	R^2	SE	CI	Pvalue
<500	-0.1393	-0.0079	1.8788	0.0573	0.1451	$r_b \pm 0.3347$	0.1826
]500-1000]	0.0752	0.0044	0.04	0.7718	0.0092	$r_b \pm 0.0194$	<0.00001
]1000-2000]	0.0295	0.0017	0.0663	0.5337	0.0047	$r_b \pm 0.0095$	<0.00001
]2000-3000]	0.0213	0.0013	0.0676	0.5892	0.0019	$r_b \pm 0.0039$	<0.00001
]3000-4000]	0.0195	0.0012	0.0618	0.7129	0.0013	$r_b \pm 0.0025$	<0.00001
]4000-5000]	0.0171	0.001	0.0586	0.7727	0.001	$r_b \pm 0.0019$	<0.00001
>5000	0.0107	0.0006	0.7817	0.2473	0.0027	$r_b \pm 0.0052$	<0.00001

Table S9. Significant t-test comparison of dataset D1 drop-off rate vs its corresponding drop-off rate of GO subset: Column 1: Gene Ontology ID (see Supplementary Table 1 for the respective GO IDs). Column 2: Pvalue: Null Hypothesis drop-off rate is not different from the main Dataset

GO	Pvalue
D1 vs GO:0000462	<0.00001
D1 vs GO:0000466	0.0556
D1 vs GO:0002181	0.0627
D1 vs GO:0003723	0.0414
D1 vs GO:0005840	0.0375
D1 vs GO:0006364	0.1046
D1 vs GO:0006396	<0.00001
D1 vs GO:0006406	0.0197
D1 vs GO:0006412	0.0088
D1 vs GO:0006950	<0.00001
D1 vs GO:0007049	0.0012
D1 vs GO:0009651	<0.00001
D1 vs GO:0015934	0.0013
D1 vs GO:0016458	<0.00001
D1 vs GO:0022625	<0.00001
D1 vs GO:0022626	0.0048
D1 vs GO:0022627	0.031
D1 vs GO:0022857	<0.00001
D1 vs GO:0030490	0.0001
D1 vs GO:0030687	<0.00001
D1 vs GO:0042254	0.003
D1 vs GO:0042274	0.0868
D1 vs GO:0042255	0.4622
D1 vs GO:0003735	0.0843
D1 vs GO:0003743	0.0704
D1 vs GO:0030684	<0.00001
D1 vs GO:0044249	<0.00001
D1 vs GO:0006457	0.005
D1 vs GO:0030686	<0.00001
D1 vs GO:0008135	0.0136
D1 vs GO:0030529	0.0526
D1 vs GO:0015935	0.0018

Table S10. Significant t-test comparison of dataset D2 drop-off rate vs its corresponding drop-off rate of GO subset: Column 1: Gene Ontology ID (see Supplementary Table 1 for the respective GO IDs). Column 2: Pvalue: Null Hypothesis drop-off rate is not different from the main Dataset

GO	Pvalue
D2 vs GO:0000462	<0.00001
D2 vs GO:0000466	<0.00001
D2 vs GO:0002181	0.0001
D2 vs GO:0003723	0.0012
D2 vs GO:0005840	0.418
D2 vs GO:0006364	<0.00001
D2 vs GO:0006396	0.0192
D2 vs GO:0006406	<0.00001
D2 vs GO:0006412	<0.00001
D2 vs GO:0006950	<0.00001
D2 vs GO:0007049	<0.00001
D2 vs GO:0009651	<0.00001
D2 vs GO:0015934	0.0001
D2 vs GO:0016458	<0.00001
D2 vs GO:0022625	<0.00001
D2 vs GO:0022626	0.5
D2 vs GO:0022627	0.1566
D2 vs GO:0022857	<0.00001
D2 vs GO:0030490	<0.00001
D2 vs GO:0030687	<0.00001
D2 vs GO:0042254	<0.00001
D2 vs GO:0042274	<0.00001
D2 vs GO:0042255	0.007
D2 vs GO:0003735	<0.00001
D2 vs GO:0003743	<0.00001
D2 vs GO:0030684	<0.00001
D2 vs GO:0044249	<0.00001
D2 vs GO:0006457	0.0135
D2 vs GO:0030686	0.009
D2 vs GO:0008135	0.0046
D2 vs GO:0030529	0.0065
D2 vs GO:0015935	<0.00001

Table S11. Significant t-test comparison of the drop-off rate of each dataset (D1, and D2) vs the drop-off rate of the corresponding gene length subset of the dataset. Column 1: Gene Length subset. Column 2: Pvalue: Null Hypothesis that the drop-off rate is not different from the main Dataset

D1	
Gene Length	Pvalue
D1 vs]0-500]	0.1843
D1 vs]500-1000]	<0.00001
D1 vs]1000-2000]	<0.00001
D1 vs]2000-3000]	<0.00001
D1 vs]3000-4000]	<0.00001
D1 vs]4000-5000]	<0.00001
D1 vs > 5000	<0.00001
**D2**	
Gene Length	Pvalue
D2 vs]0-500]	0.2309
D2 vs]500-1000]	<0.00001
D2 vs]1000-2000]	<0.00001
D2 vs]2000-3000]	<0.00001
D2 vs]3000-4000]	<0.00001
D2 vs]4000-5000]	<0.00001
D2 vs > 5000	<0.00001

Table S12. Significant t-test comparison of the drop-off rate of each dataset (D3, and D4) vs the drop-off rate of the corresponding gene length subset of the dataset. Column 1: Gene Length subset. Column 2: Pvalue: Null Hypothesis that the drop-off rate is not different from the main Dataset

D3	
Gene Length	Pvalue
D3 vs]0-500]	0.0853
D3 vs]500-1000]	<0.00001
D3 vs]1000-2000]	<0.00001
D3 vs]2000-3000]	<0.00001
D3 vs]3000-4000]	<0.00001
D3 vs]4000-5000]	<0.00001
D3 vs > 5000	0.3566
**D4**	
Gene Length	Pvalue
D4 vs]0-500]	0.4768
D4 vs]500-1000]	<0.00001
D4 vs]1000-2000]	<0.00001
D4 vs]2000-3000]	<0.00001
D4 vs]3000-4000]	<0.00001
D4 vs]4000-5000]	<0.00001
D4 vs > 5000	<0.00001

Table S13. Significant t-test comparison of the drop-off rate of each dataset (D5, D6, D7, and D8) vs the drop-off rate of the corresponding gene length subset of the dataset. Column 1: Gene Length subset. Column 2: Pvalue: Null Hypothesis that the drop-off rate is not different from the main Dataset

D5	
Gene Length	Pvalue
D5 vs]0-500]	0.1005
D5 vs]500-1000]	<0.00001
D5 vs]1000-2000]	<0.00001
D5 vs]2000-3000]	<0.00001
D5 vs]3000-4000]	<0.00001
D5 vs]4000-5000]	<0.00001
D5 vs > 5000	0.1067
**D6**	
Gene Length	Pvalue
D6 vs]0-500]	0.1927
D6 vs]500-1000]	<0.00001
D6 vs]1000-2000]	<0.00001
D6 vs]2000-3000]	<0.00001
D6 vs]3000-4000]	<0.00001
D6 vs]4000-5000]	<0.00001
D6 vs > 5000	0.0473
**D7**	
Gene Length	Pvalue
D7 vs]0-500]	0.1528
D7 vs]500-1000]	<0.00001
D7 vs]1000-2000]	<0.00001
D7 vs]2000-3000]	<0.00001
D7 vs]3000-4000]	<0.00001
D7 vs]4000-5000]	0.0003
D7 vs > 5000	0.1476
**D8**	
Gene Length	Pvalue
D8 vs]0-500]	0.1826
D8 vs]500-1000]	<0.00001
D8 vs]1000-2000]	0.0001
D8 vs]2000-3000]	<0.00001
D8 vs]3000-4000]	0.0001
D8 vs]4000-5000]	0.0006
D8 vs > 5000	0.2162

Table S14. Drop-off rate for Datasets D1 to D8 using Binsize =100 and 25. Column 1: Datasets ID. Column 2: Drop-off rate per bin (r_b). Column 3: Drop-off rate per codon (r_c). Column 4: $RMSE$. Column 5: coefficient of determination (R^2). Column 6: Standard Error Estimate (SE). Column 7: Confidence Interval 95%. Column 8: p-value resulting from the t-test (null hypothesis: $r_b = 0$) performed according to Equation 6.

Dataset	Drop-off (r_b)	Dropoff per codon (r_c)	RMSE	R^2	SE	CI	Pvalue
			binsize =100				
D1	0.0097	0.0003	0.0084	0.5967	0.001	r_b±0.002	<0.00001
D2	0.02	0.0006	0.0211	0.7159	0.0018	r_b±0.0036	<0.00001
D3	0.0192	0.0006	0.0579	0.4588	0.0029	r$_b$±0.0057	<0.00001
D4	0.0138	0.0004	0.0204	0.5539	0.0007	r_b±0.0013	<0.00001
D5	0.0203	0.0006	0.6719	0.0756	0.0038	r$_b$±0.0075	<0.00001
D6	0.0169	0.0005	0.3845	0.0905	0.0038	r_b±0.0074	<0.00001
D7	0.0235	0.0007	0.7046	0.0949	0.0078	r_b± 0.0154	0.0015
D8	0.0217	0.0006	0.5529	0.102	0.0055	r_b±0.011	0.0001
			binsize =25				
Gene	Drop-off (r_b)	Dropoff per codon (r_c)	RMSE	R^2	SE	CI	Pvalue
D1	0.003	0.0004	0.0751	0.2095	0.0004	r_b± 0.0008	<0.00001
D2	0.0059	0.0007	0.1277	0.3672	0.0003	r_b± 0.0006	<0.00001
D3	0.0053	0.0006	0.1773	0.2558	0.0005	r$_b$±0.001	<0.00001
D4	0.0044	0.0005	0.1454	0.225	0.0002	r_b± 0.0004	<0.00001
D5	0.0018	0.0002	0.4023	0.0174	0.001	r$_b$±0.002	0.036
D6	0.0023	0.0003	0.2884	0.0372	0.001	r_b±0.002	0.0128
D7	0.0032	0.0004	0.4543	0.0461	0.0009	r_b±0.0018	0.0002
D8	0.0034	0.0004	0.3596	0.0633	0.001	r_b±0.002	0.0005

Table S15. Drop-off rate for control datasets D1, and D2 and separate genes as a special case: Column 1: Gene Name. Column 2: Drop-off rate per bin (r_b). Column 3: Drop-off rate per codon (r_c). Column 4: $RMSE$. Column 5: coefficient of determination (R^2). Column 6: Standard Error Estimate (SE). Column 7: Confidence Interval 95%. Column 8: p-value resulting from the t-test (null hypothesis: $r_b = 0$) performed according to Equation 6..

			D1				
Gene	Drop-off (r_b)	Dropoff per codon (r_c)	RMSE	R^2	SE	CI	Pvalue
MHS2	0.0326	0.0019	0.2171	0.5778	0.0037	r_b± 0.0075	<0.00001
MLH1	0.0668	0.0039	19.7502	0.0399	0.0488	r_b±0.0984	0.0892
			D2				
Gene	Drop-off (r_b)	Dropoff per codon (r_c)	RMSE	R^2	SE	CI	Pvalue
MHS2	0.0498	0.0029	0.1671	0.8061	0.0033	r_b±0.0065	<0.00001
MLH1	0.085	0.0049	2.9649	0.3097	0.0189	r_b± 0.0381	<0.00001

Table S16. Drop-off rate for treatment datasets D3, and D4 and separate genes as a special case: Column 1: Gene Name. Column 2: Drop-off rate per bin (r_b). Column 3: Drop-off rate per codon (r_c). Column 4: $RMSE$. Column 5: coefficient of determination (R^2). Column 6: Standard Error Estimate (SE). Column 7: Confidence Interval 95%. Column 8: p-value resulting from the t-test (null hypothesis: $r_b = 0$) performed according to Equation 6.

			D3				
Gene	Drop-off (r_b)	Dropoff per codon (r_c)	RMSE	R^2	SE	CI	Pvalue
MHS2	0.0508	0.003	0.4033	0.6418	0.0051	r_b±0.0102	<0.00001
MLH1	0.0354	0.0021	11.5249	0.0196	0.0373	r_b±0.0751	0.1741
			D4				
Gene	Drop-off (r_b)	Dropoff per codon (r_c)	RMSE	R^2	SE	CI	Pvalue
MHS2	0.044	0.0026	0.2331	0.6999	0.0039	r_b±0.0077	<0.00001
MLH1	0.047	0.0028	0.6852	0.3727	0.0091	r_b±0.0183	<0.00001

Table S17. Gene Length subset sizes. Column 1 is Gene Length subset. Column 2 is set size.

Gene Length Subset	Set Size
]0-500]	1573
]500-1000]	1547
]1000-2000]	2213
]2000-3000]	801
]3000-4000]	282
]4000-5000]	119
>5000	77

S3. SUPPLEMENTARY FIGURES

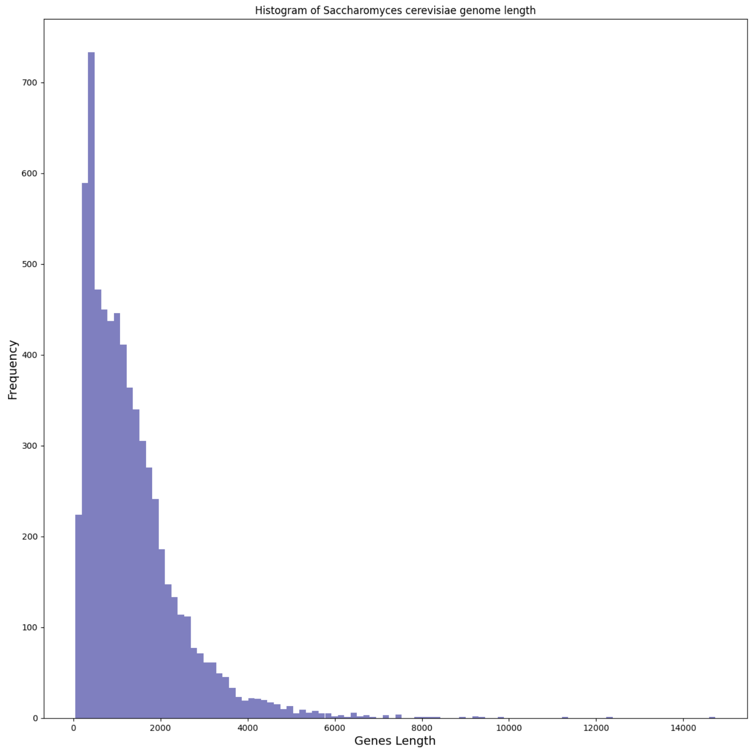

Figure S1. Saccharomyces cerevisiae Gene Length Distribution

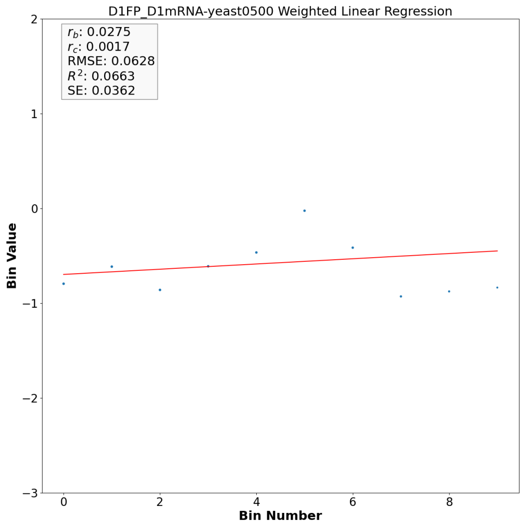

Figure S2. D1 (Synthetic Defined): Subset with Gene Length [0-500[

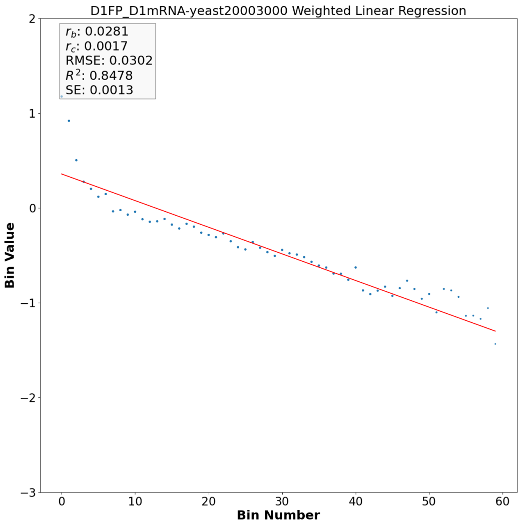

Figure S3. D1 (Synthetic Defined): Subset with Gene Length]2000-3000]

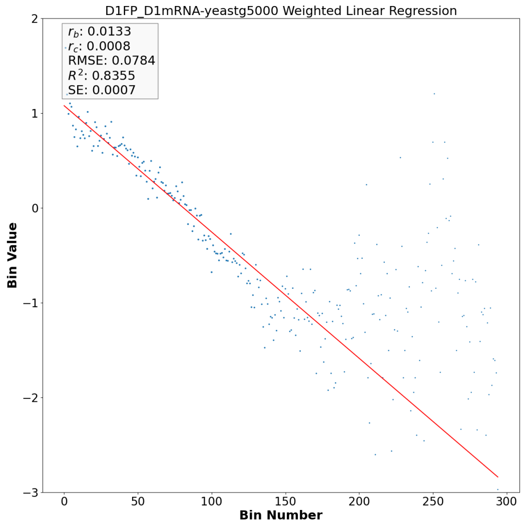

Figure S4. D1 (Synthetic Defined): Subset with Gene Length greater than 5000

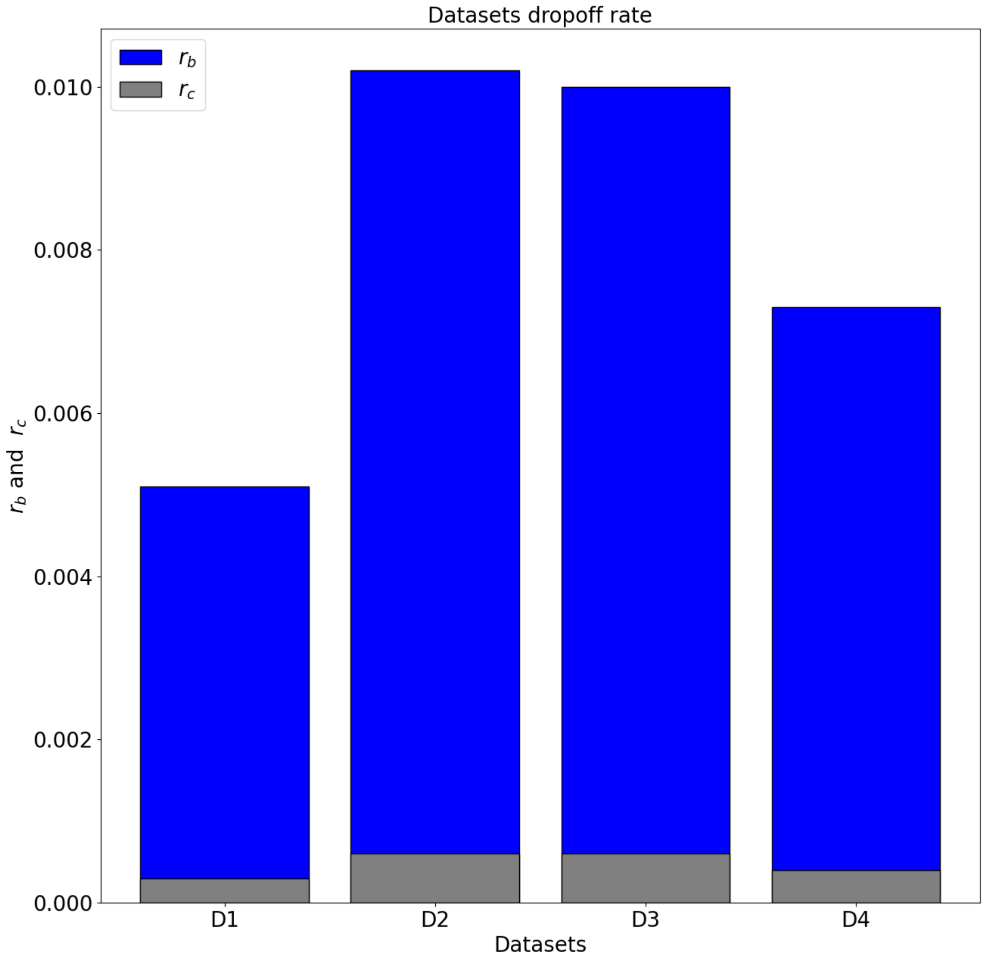

Figure S5. Dropoff rate per bin and Dropoff per codon for the four main Datasets

S4. A CASE STUDY: MSH2 AND MLH1 GENES

Ribofilio can be used to estimate the drop-off rate on a selected genes. Here, we used ribofilio to estimate the drop-off rate of genes MSH2 and MLH1 in yeast which are homologous to human gene MSH2 and MLH1 respectively (1, 6),(1, 6). MSH2 has been used to study Lynch syndrome, breast cancer, and ovarian cancer (2, 7). Yeast MLH1 has been used to study colorectal cancer (3, 5). We studied the drop-off rate of both MSH2 and MLH1 in both main datasets D1 and D2. Table S15 shows the drop-off rates of MSH2 and MLH1 in main datasets D1 and D2. Table S16 shows the drop-off rates of MSH2 and MLH1 in main datasets D3 and D4.

REFERENCES

1. J Michael Cherry, Caroline Adler, Catherine Ball, Stephen A Chervitz, Selina S Dwight, Erich T Hester, Yankai Jia, Gail Juvik, TaiYun Roe, Mark Schroeder, et al. Sgd: Saccharomyces genome database. *Nucleic acids research*, 26(1):73–79, 1998.
2. Mira Goldberg, Kathleen Bell, Melyssa Aronson, Kara Semotiuk, Greg Pond, Steven Gallinger, and Kevin Zbuk. Association between the lynch syndrome gene msh2 and breast cancer susceptibility in a canadian familial cancer registry. *Journal of Medical Genetics*, 54(11):742–746, 2017.
3. Sigurdis Haraldsdottir, Heather Hampel, Christina Wu, Daniel Y Weng, Peter G Shields, Wendy L Frankel, Xueliang Pan, Albert De La Chapelle, Richard M Goldberg, and Tanios Bekaii-Saab. Patients with colorectal cancer associated with lynch syndrome and mlh1 promoter hypermethylation have similar prognoses. *Genetics in medicine*, 18(9):863–868, 2016.
4. Michael Hilton, Jonathan Bell, and Darko Marinov. A large-scale study of test coverage evolution. In *Proceedings of the 33rd ACM/IEEE International Conference on Automated Software Engineering*, pages 53–63, 2018.
5. Tomoyuki Momma, Kenji Gonda, Yoshinori Akama, Eisei Endo, Daisuke Ujiie, Shotaro Fujita, Yuko Maejima, Shoichiro Horita, Kenju Shimomura, Shigehira Saji, et al. Mlh1 germline mutation associated with lynch syndrome in a family followed for more than 45 years. *BMC Medical Genetics*, 20(1):1–6, 2019.
6. Marek S Skrzypek and Jodi Hirschman. Using the saccharomyces genome database (sgd) for analysis of genomic information. *Current protocols in bioinformatics*, 35(1):1–20, 2011.
7. Aung Ko Win, Noralane M Lindor, and Mark A Jenkins. Risk of breast cancer in lynch syndrome: a systematic review. *Breast Cancer Research*, 15(2):1–9, 2013.
